# Controlled Delivery of H_2_O_2_: A Three-Enzyme Cascade Flow Reactor for Peroxidase-Catalyzed Reactions

**DOI:** 10.1021/acssuschemeng.4c03220

**Published:** 2024-06-27

**Authors:** Simin Arshi, Ketan Madane, Kim Shortall, Goran Hailo, Julia Alvarez-Malmagro, Xinxin Xiao, Katarzyna Szymanńska, Serguei Belochapkine, Vivek V. Ranade, Edmond Magner

**Affiliations:** † Department of Chemical Sciences, Bernal Institute, University of Limerick, Limerick V94 T9PX, Ireland; ‡ Department of Chemistry, Technical University of Denmark, Kongens Lyngby 2800, Denmark; § Department of Chemical Engineering and Process Design, Silesian University of Technology, Gliwice 44-100, Poland

**Keywords:** flow reactor, enzyme cascade, peroxygenase-catalyzed reactions, hydrogen peroxide

## Abstract

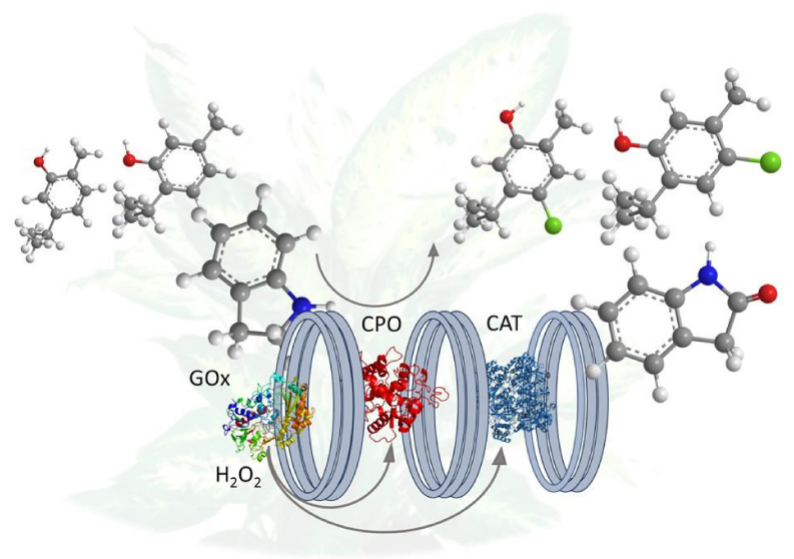

Peroxidases are promising catalysts for oxidation reactions, yet their practical utility has been hindered by the fact that they require hydrogen peroxide (H_2_O_2_), which at high concentrations can cause deactivation of enzymes. Practical processes involving the use of peroxidases require the frequent addition of low concentrations of H_2_O_2_. *In situ* generation of H_2_O_2_ can be achieved using oxidase-type enzymes. In this study, a three-enzyme cascade system comprised of a H_2_O_2_ generator (glucose oxidase (GOx)), H_2_O_2_-dependent enzymes (chloroperoxidase (CPO) or horseradish peroxidase (HRP)), and a H_2_O_2_ scavenger (catalase (CAT)) was deployed in a flow reactor. Immobilization of the enzymes on a graphite rod was achieved through electrochemically driven physical adsorption, followed by cross-linking with glutaraldehyde. Modeling studies indicated that the flow in the reactor was laminar (Reynolds number, *R*_*e*_ < 2000) and was nearly fully developed at the midplane of the annular reactor. Immobilized CAT and GOx displayed good stability, retaining 79% and 84% of their initial activity, respectively, after three cycles of operation. Conversely, immobilized CPO exhibited a considerable reduction in activity after one use, retaining only 30% of its initial activity. The GOx-CAT-GRE system enabled controlled delivery of H_2_O_2_ in a more stable manner with a 4-fold enhancement in the oxidation of indole compared to the direct addition of H_2_O_2_. Using CPO in solution coupled with GOx-CAT-GRE yields of 90% for the oxidation of indole to 2-oxyindole and of 93% and 91% for the chlorination of thymol and carvacrol, respectively.

## Abstract 2

A three-enzyme cascade reactor was developed with the ability to add hydrogen peroxide for the selective oxidation of substrates.

## 1 Introduction

Peroxidases possess the capability to catalyze a wide variety of oxidation reactions, ranging from reactions driven by radical mechanisms, to oxygen insertion into C–H bonds and two-electron substrate oxidation.^1,2^ Peroxidases employ H_2_O_2_ directly to generate catalytically active oxyferryl-heme species, which subsequently continues the reaction pathway.^1−4^ Chloroperoxidase (CPO) is an extracellular heme glycoprotein that exhibits halogenase, catalase (CAT), and peroxidase activity.^5^ The ability of CPO to act as a catalyst in a variety of synthetically useful reactions, including halogenation,^6^ heteroatom oxidation (*N*- and *S*-oxidation),^7^ epoxidation,^8^ and the oxidation of alcohols,^9,10^ with high regioselectivity and enantioselectivity is of interest. Zaks and Dodds investigated the substrate specificity of CPO in a series of oxidation reactions and demonstrated that straight-chain aliphatic and cyclic cis-olefins could undergo epoxidation in a highly stereoselective manner, while aliphatic and aromatic alcohols were oxidized to their corresponding aldehydes and acids.^8^ The oxidation of indole is of interest as oxygenated indole derivatives are used in the treatment of several diseases including cancer, diabetes, and viral infections.^14^ In the presence of chloroperoxidase (CPO), indole is oxidized by H_2_O_2_ to 2-oxindole as the main product. Despite the potential of peroxygenases, their practical application is hindered by poor operational stability toward H_2_O_2_, leading to oxidative degradation of the catalytic heme moiety. However, the requirement for H_2_O_2_ as a cosubstrate, particularly at high concentrations, can hamper the use of peroxidases.^11^ In general, this problem can be tackled by *in situ* production of H_2_O_2_ using electrochemical,^12^ photochemical,^13^ photoelectrochemical,^14^ or enzymatic methods.^15^ Getrey et al. reported the use of free CPO for the conversion of thymol and carvacrol into their corresponding halogenated forms (90% conversion) using H_2_O_2_ produced at a rate of 0.53 μmol min^–1^ cm^–2^ by a gas diffusion electrode.^6^ Enzymatic methods can be used to produce H_2_O_2_ under mild conditions. Glucose oxidase (GOx) oxidizes glucose to d-glucono-δ-lactone and hydrogen peroxide, with high stability toward H_2_O_2_.^16^ The bienzymatic cascade, CPO and GOx, has been used for the batch oxidation reactions such as the selective oxidation of sulfides,^7^ alcohols,^9^ and the epoxidation of alkenes.^17^ van de Velde et al. used a combination of CPO with GOx immobilized in polyurethane foams. Upon coimmobilization with glucose oxidase, the turnover frequencies (TOFs) of chloroperoxidase for the oxidation of thioanisole and cis-2-heptene were 500 and 50 min^–1^, respectively, in the presence of 30% (v:v) tert-butyl alcohol (t-BuOH).^17^ Choi et al. reported the *in situ* production of H_2_O_2_ at a rate 0.37 mM h^–1^ using a flavin-SWNT-based photoelectrochemical platform that was used for peroxygenase catalyzed reactions such as the oxidation of indole by CPO, with turnover number (TON molproduct mol enzyme^–1^) and a space time yield (STY, g L^–1^ d^–1^) of 4,900 ± 340 and 0.88 ± 0.06, respectively.^14^ As H_2_O_2_ is a potent oxidant;^18^ its concentration needs to be controlled to avoid oxidation of other substances and to limit enzyme deactivation. Catalase can decompose H_2_O_2_ into water and oxygen and can be used to control the concentration of H_2_O_2_ and maintain the activity of peroxidases.^19^

The immobilization of enzymes can improve system efficiency by enabling the reuse of enzymes and increasing productivity and stability.^20,21^ Furthermore, immobilization allows enzymes to be used in continuous operation, but not all immobilization methods result in active and stable enzymes inside the flow reactor.^22,23^ Electrochemical immobilization of enzymes can be used for controlled and stable immobilization of enzymes.^24,25^ In a recent study, we immobilized GOx on the surfaces of electrodes modified with conductive polymers, silica films, and diazonium linkers for the controlled production of H_2_O_2_. The immobilization technique had an impact on the rate of H_2_O_2_ production and the stability of modified electrodes. GOx encapsulated in polypyrrole (PPy) showed excellent stability (7 h) with a rate of production of 602 ± 57 μM h^–1^.^26^ An electrochemically induced adsorption of GOx can be used for the immobilization in a simple manner. For example, Toit et al. immobilized GOx on porous gold electrodes by scanning (6 cycles) over the potential range 0.42 to 0.6 V at pH 7. The isoelectric point (pI) of GOx has been reported 4.2,^27^ and the enzyme is negatively charged at pH 7.^28^ The application of a potential more positive than the potential of zero charge (*E*_pzc_) will enhance electrostatic attraction between the electrode surface and GOx.^29,30^

A range of supports have been utilized to immobilize CPO; these include electro-deposited polymer film electrodes,^31^ mesoporous silicates,^32^ and magnetic nanoparticles.^33^ Considerable attention has been given to the immobilization of CPO on mesoporous materials such as MCM-48, SBA-15, SBA-16, and mesocellular foam (MCF).^34^ SBA-15 and MCF possessed large pore sizes (average pore diameters of 70 and 150 Å, respectively) and were used to immobilize CPO.^35^ Bakker et al. used polyurethane foams to encapsulate CPO covalently.^36^ The immobilized enzyme retained its activity, and no leaching was observed under the reaction conditions. The immobilization of CPO has been reported on epoxy-modified silica gel and aldehyde-functionalized sol–gel glass.^37,38^ He et al.^33^ described the immobilization of CPO using the carbohydrate-binding lectin protein, concanavalin A (ConA) on silica-coated magnetic nanoparticles (MNPs), with a retention of 80% of initial activity after eight cycles, and 70% ee in the asymmetric synthesis of modafinil.^39,40^

Tubular and annular flow reactors have been used for a range of reactions such as oxidation,^41,42^ hydrolysis,^43,44^ and transesterification reactions.^45,46^ Parameters, such as the flow rate and reactor dimensions (ranging from centimeter^47,48^ to meter^49−51^) have been examined in detail to understand the conditions of the reaction environment in the reactor.^52−55^ A detailed critical review and in-depth analysis of flow modeling in such reactors and the challenges in designing and evaluating their performance was provided by Rivera et al.^56^ Catañeda et al. reviewed the computational methods and approaches used to assess a range of commercially available reactors.^54^ The properties of annular flow reactors have been evaluated in detail both computationally and experimentally.^57−60^

**Scheme 1:**
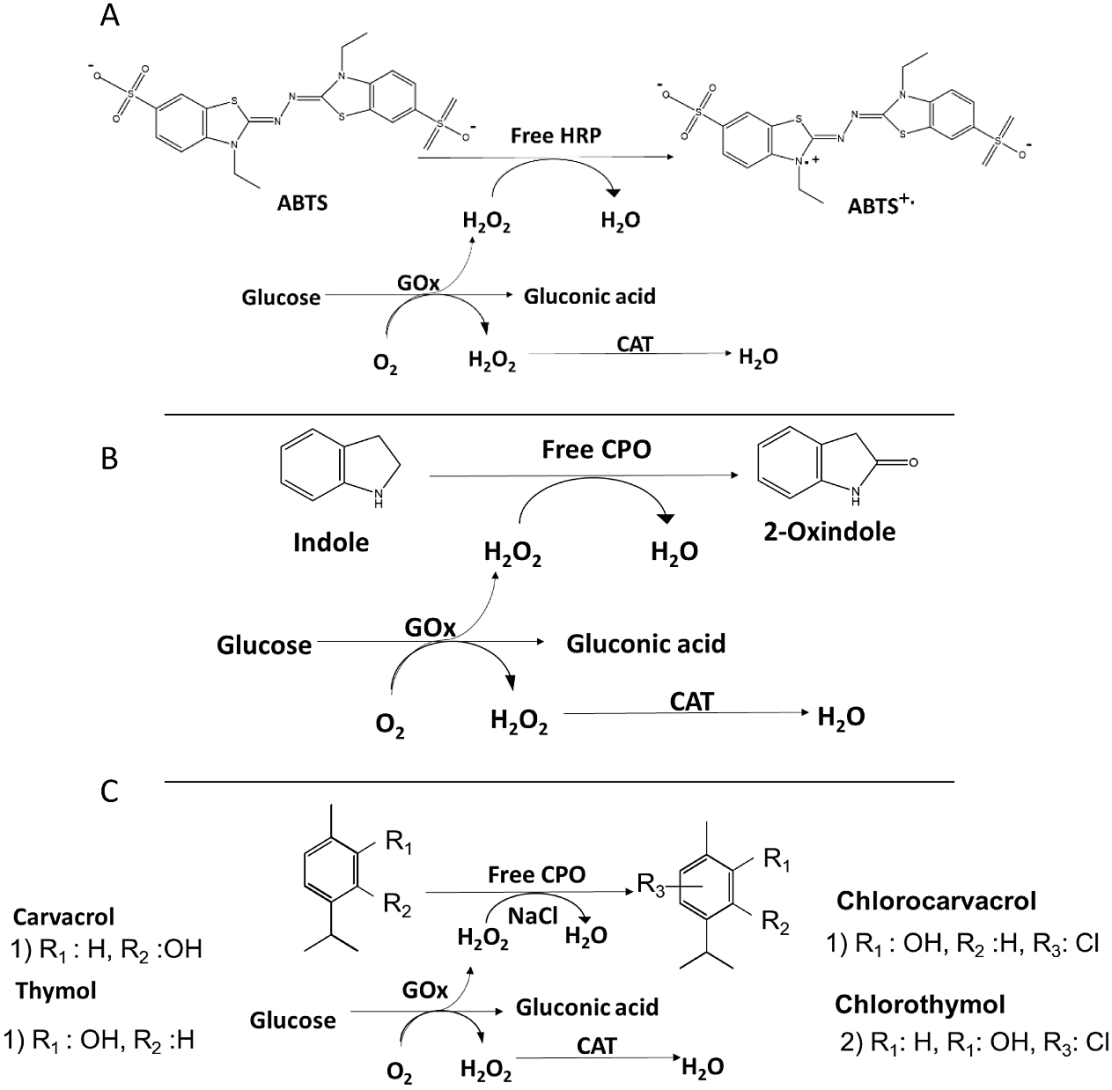
Schematic Diagrams of the Reactions (A) The oxidation of ABTS by the three-enzyme cascade GOx-HRP-CAT, (B) the selective oxidation of indole, and (C) halogenation of thymol and carvacrol catalyzed by the three-enzyme cascade GOx-CPO-CAT in the flow system.

In this study, a three-enzyme cascade (GOx-CPO/HRP-CAT) was developed to control the concentration of hydrogen peroxide within the flow system and to facilitate oxidation reactions. Moreover, a simple one-step electrochemical method was developed for the electrochemical immobilization of enzymes on the surface of a graphite rod. GOx was used for the *in situ* production of H_2_O_2_ and was coupled with H_2_O_2_-dependent enzymes, CPO or HRP, and CAT as a H_2_O_2_ scavenger. Each enzyme was immobilized onto an individual graphite rod and was subsequently connected to form a series of three reactors. The system was used to perform the oxidation of indole and ABTS and for the halogenation of thymol and carvacrol (Scheme 1). The combination of GOx and CAT resulted in an improved yield of 2-oxindole compared to the process where H_2_O_2_ was added externally. Computational fluid dynamics (CFD) was used to investigate the performance of the annular flow reactor.^24^ The flow, mixing, and residence time distribution (RTD) characteristics were examined to provide a basis to further develop, optimize, and translate these results to practice.

## 2 Experimental Section

### 2.1 Reagents and Materials

Dibasic and monobasic sodium phosphate, ethanol, potassium chloride, d-(+)-glucose, glucose oxidase (GOx) from *Aspergillusniger* (50 000 U mg^–1^), peroxidase from horseradish (HRP), catalase from bovine liver (CAT), chloroperoxidase from *Caldariomycesfumago* (CPO), 2,2’-azino-bis(3-ethylbenzothiazoline-6-sulfonic acid) diammonium salt (ABTS), hydrogen peroxide (H_2_O_2_), glutaraldehyde, potassium chloride, thioanisole, thymol, carvacrol potassium hydroxide, Sepharose CL-6B, sodium borohydride (≥99%), glycidol (96%), sodium periodate (≥99%), monochlorodimedon (MCD), tert-butanol (t-BuOH), sodium carbonate, and sodium bicarbonate were obtained from Sigma–Aldrich, Ireland, Ltd. Cis-2-heptene was obtained from Santa Cruz Biotechnology. All solutions were prepared with deionized water from an Elgastat maxima-HPLC (Elga Purelab Ultra, UK). All chemicals were of reagent-grade purity.

Electrochemical experiments were performed with a CHI630A potentiostat (CH Instruments, Austin, Texas). A conventional three-electrode cell was used with a fine extruded graphite rod (GRE, 4.77 mm) (Graphite Store) stainless-steel mesh (4 × 3 cm^2^) and Ag/AgCl (4 M KCl) as the working, counter, and reference electrodes, respectively. Prior to electrode modification, GREs were polished with sandpaper (P2000), cleaned by sonication in a solution of water-ethanol-acetone (1:1:1) for 9 min, and dried in the oven. Absorbance measurements were recorded on a Cary 60 UV–vis spectrophotometer (Agilent, USA). An Agilent 1260 HPLC (Agilent Technologies, Waldbronn, Germany) with a UV–vis photodiode array detector with a reverse-phase column (Zorbax RX-C18 250 mm × 4.6 mm, 5 μm) was used for analysis.

### 2.2 Immobilization of Enzymes on GRE

**Scheme 2:**
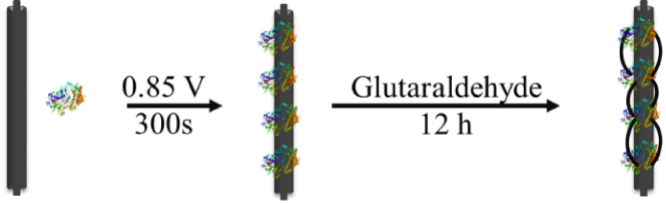
Schematic Diagrams of Enzyme Immobilization on GRE

Enzyme immobilization was performed by adapting a previously published approach (Scheme 2).^61^ GREs were immersed in a solution of the enzyme (HRP 0.4 mg ml^–1^; CPO 3700 U; CAT 2 or 4 mg ml^–1^ and GOx 1 mg ml^–1^) in NaPi (pH 6, 0.1 M), and a potential of 0.85 V (vs Ag/AgCl) was applied for 300 s at 20 °C. The enzyme-modified GREs were immersed in glutaraldehyde (1% in NaPi, pH 6, 0.1 M) for 12 h at 4 °C. The modified GREs are denoted as HRP-GRE, CPO-GRE, CAT-GRE, and GOx-GRE. Immobilization of catalase in polypyrrole (CAT-PY-GRE) was performed according to our previous study with some changes.^26^ A constant potential (0.85 V vs Ag/AgCl) was applied for 300 s in a degassed solution of Py (0.2 M) and CAT (2 mg ml^–1^) in NaPi (0.1 M, pH 7) at 20 °C. Enzymes were immobilized on GRE with surface areas of 5.08 and 1.4 cm^2^ in the flow and batch reactors, respectively. Modified GREs were stored at 4 °C under 100% humidity until further use. Before use, all modified GREs were placed in a solution of NaPi (0.1 M, pH 5) for 1 h at room temperature.

### 2.3 Surface Charge Distribution of Enzymes

The surface charge distribution was obtained through electrostatic calculations carried out using the adaptive Poisson–Boltzmann solver (APBS) after conversion of the PDB file to a PQR file using PDB2PQR (https://server.poissonboltzmann.org/pdb2pqr) with input of the desired pH. The results were visualized using PyMOL,^62^ with PyMOL APBS tools. PBD IDs utilized: GOx (1CF3), HRP (2YLJ), CPO (1CPO). and CAT (1TGU).

### 2.4 Flow Reactor

An annular flow reactor was designed to incorporate GRE, as described in our previous study.^26^ An Instech P720 peristaltic pump was used to pump the solution through the channel, and the solution was pumped into the channel at a flow rate of 0.08 mL min^–1^.

### 2.5 Activity of GOx-GRE, CPO-GRE, HRP-GRE, and CAT-GRE on GRE in Batch and Flow Reactors

The catalytic activity of modified GREs was initially investigated in a batch mode by immersing HRP-GRE in ABTS 0.1 mM, H_2_O_2_ 0.05 mM; CPO-GRE in thioanisole ca. 55 μM, H_2_O_2_ 0.2 mM; CAT-GRE and CAT-PY-GRE in H_2_O_2_ 0.2 mM; and GOx-GRE in glucose 50 mM for 30 min. All solutions were prepared in NaPi (0.1 M, pH 5) or 20% t-ButOH/NaPi (0.1 M, pH 5). The activity of the immobilized enzymes was measured at 30 min intervals using a fresh reaction solution (1 mL). The concentration of thioanisole was determined by monitoring the absorbance (255 nm) at 25 °C. The concentration of H_2_O_2_ was determined using an HRP assay with ABTS. The assay conditions were 0.54 mM ABTS and 0.01 mg ml^–1^ HRP in NaPi (0.1 M, pH 7) at 420 nm^26^ and 25 °C. CAT-GRE coupled with GOx-GRE were investigated in the flow reactor using 3 mL glucose 50 mM in NaPi (0.1 M, pH 5) or 20% t-ButOH/NaPi (0.1 M, pH 5). The activity of CPO-GRE was investigated using a solution of thioanisole (0.1 mM) and H_2_O_2_ (0.2 mM) in 20% t-ButOH/NaPi (0.1 M, pH 5), with a total volume of 3 mL.

### 2.6 Residual Activity of GOx-GRE, CAT-GRE, and CPO-GRE in the Flow Reactor

Prior to use, the initial catalytic activity of the modified electrodes was determined by immersion in the assay solution (2 mL, 5 min). After use in the reactor, the residual catalytic activity was determined in a fresh assay solution under the same conditions.

### 2.7 Assay of Activity of CPO in Solution

The monochlorodimedon (MCD) assay^63^ was carried out to determine the enzyme activity of immobilized and free CPO. The final concentrations of MCD, KCl, and free CPO in the quartz cuvette were 0.1 mM, 20 mM, and 0.056 μg ml^–1^, respectively, in NaPi (0.1 M, pH 5). The reaction was initiated with the addition of 60 μL of 0.09 M H_2_O_2_. The specific activity of free CPO was 751 ± **9.7** μmol min^–1^ mg^–1^.

### 2.8 Enzymatic Reactions and Product Quantification

The rate of oxidation of ABTS was monitored using HRP (0.2 mg ml^–1^) in a solution containing 50 mM glucose and 1 mM ABTS in NaPi (0.1 M, pH 5), and the concentration of ABTS was quantified by monitoring the absorbance at 340 nm (ε = 39.8 mM^–1^ cm^–1^, a value similar to the reported value of 36 mM^–1^ cm^–1^ .^64^ Indole oxidation was carried out using GOx/CPO/CAT-GRE or GOx/free CPO (17 U, 0.024 mg)/CAT-GRE. The reaction solution contained glucose (50 mM) and indole (1 mM) in NaPi (0.1 M, pH 5) or 20% t-BuOH, pH 5. The separation of 2-oxindole and indole was performed using HPLC with a mobile phase (MeOH and water (50:50 v/v)) at a flow rate of 0.9 mL min^–1^, a temperature of 30 °C, and using a UV detector (250 nm). Halogenation of thymol and carvacrol was performed using GOx/free CPO (17 U)/CAT-GRE. The reaction solution contained thymol or carvacrol (1 mM), NaCl (20 mM), and glucose (50 mM) in 20% t-BuOH/NaPi (0.1 M, pH 5). The production of thymol and carvacrol was followed during the reaction using HPLC with a mobile phase (80% MeOH and 20% (v/v) water) flowing at a flow rate of 1.2 mL min^–1^, a temperature of 40 °C, and using a UV detector (278 nm) and mass spectroscopy to determine the products. The reactions were quenched by the addition of methanol and cooling. All reactions were conducted at room temperature (20 °C). Samples were collected upon completion of the reaction (after 240 min). Ethyl acetate was utilized to extract the products, and the solvent was evaporated. The products were dissolved in methanol and analyzed using MS (Agilent 6530 Accurate-Mass QTOF, University of Galway): thymol or carvacrol: *m*/*z* 150 [M]^+^ and 149[M–H]^+^; chlorothymol: (negative mode) *m*/*z* 183[M-H]^+65^ and *m*/*z* 184[M]^+^;^65^ dichlorocarvacrol: (positive mode) *m*/*z* 236 [M+NH_4_]^+^.

## 3 Results and Discussion

### 3.1 Immobilization of Enzymes on GRE and their Activity in Batch Processes

**Figure 1:**
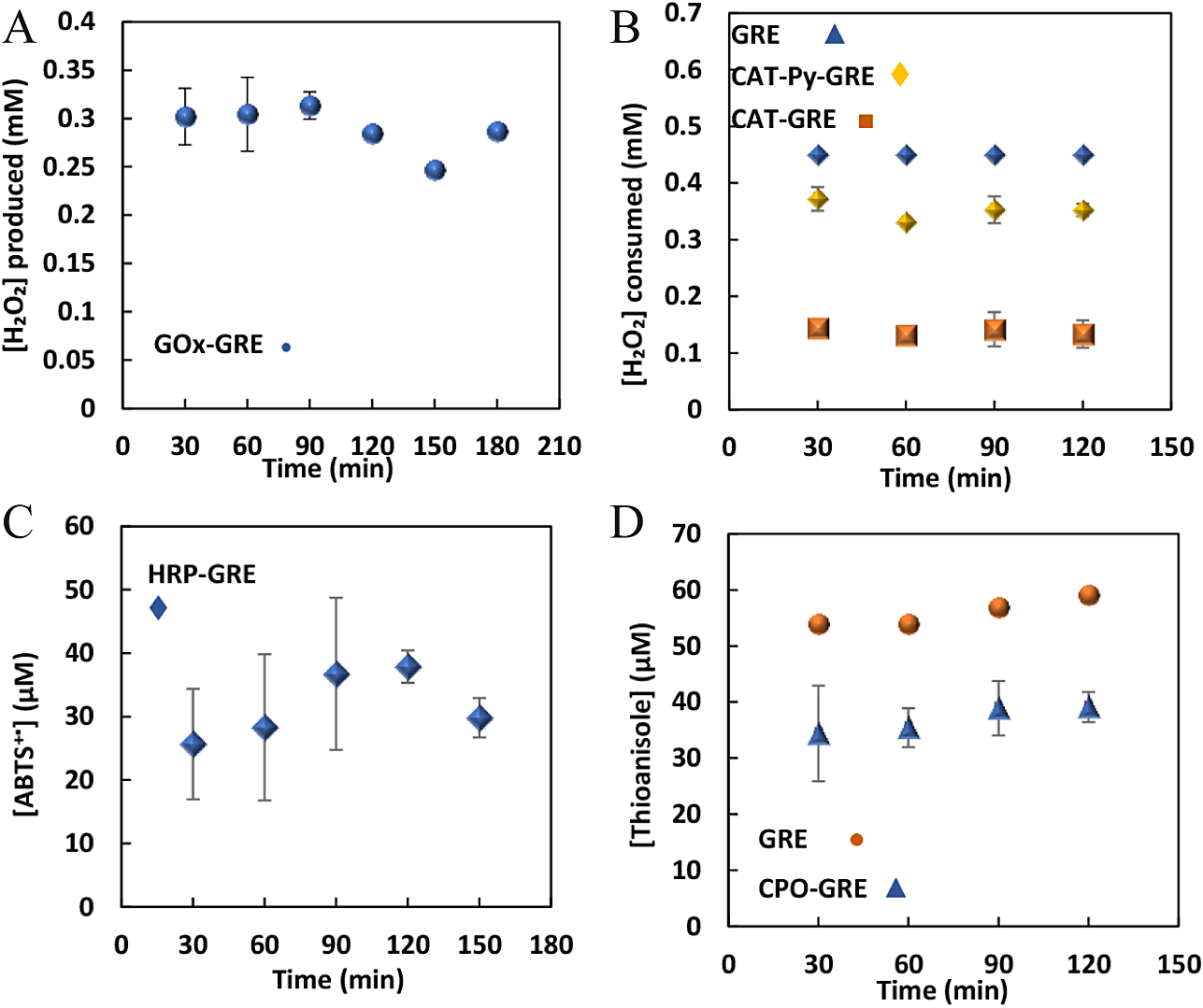
Plot of concentration of (A) H_2_O_2_ produced at GOx-GRE in the batch system; 50 mM glucose in NaPi (0.1 M, pH 5), total volume of 1 mL; (B) H_2_O_2_ consumed at CAT-Py-GRE, CAT-GRE, GRE, 0.45 mM H_2_O_2_ in NaPi (0.1 M, pH 5), a total volume of 1 mL; (C) ABTS^+■^ produced at HRP-GRE, 0.1 mM ABTS, 0.2 mM H_2_O_2_ in NaPi (0.1 M, pH 5), a total volume of 1 mL; (D) thioanisole consumed at CPO-GRE (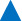), GRE (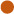); thioanisole 0.05 mM, H_2_O_2_ 0.1 mM in 20% t-ButOH/NaPi (0.1 M, pH 5), the total volume of 1 mL. Error bars indicate the standard deviation of duplicate or triplicate experiments.

GOx, CPO, HRP, and CAT were immobilized (Scheme 2) by applying a potential of 0.85 V (vs Ag/AgCl) to GREs immersed in solutions of the appropriate enzyme (1 mg ml^–1^ GOx, 0.4 mg ml^–1^ HRP, 0.34 mg ml^–1^ CPO, or 2 mg ml^–1^ CAT) in NaPi (pH 7) for 300 s. The enzymes were immobilized on the surface by electrochemically driven physical adsorption.^30^ The *E*_pzc_ of graphite is −0.237 V vs Ag/AgCl,^66^ and at an applied potential of 0.85 V vs Ag/AgCl, the electrode surface is positively charged, increasing the degree of electrostatic attraction between the electrode surface and negatively charged enzymes at pH 7. The surface charge distributions of the enzymes are shown in Figure S2. GREs were immersed in glutaraldehyde to cross-link the adsorbed protein film. Immersion of the GOx-modified electrode in a solution containing glucose 50 mM resulted in the production of 0.3 mM H_2_O_2_ (30 min) with no loss in activity in 4 consecutive cycles (ca. 2 h) (Figure 1A) indicating that no leaching had occurred. Using GOx-GRE, the rate of production of H_2_O_2_ was 2-fold higher than that obtained with GOx immobilized in polypyrrole on GRE.^26^ This difference can be ascribed to diffusional limitations associated with the presence of the polymer in the latter system.

CAT was immobilized on GRE using two separate methods: electrodeposition of PPy (CAT-PPy-GRE) and electrochemically driven physical adsorption (CAT-GRE). The activity of the immobilized enzyme was investigated by immersion in a solution of 0.45 mM H_2_O_2_. Using CAT-Py-GRE resulted in the reduction of 0.08 mM H_2_O_2_ in 30 min (Figure 1B), while CAT-GRE exhibited higher catalytic activity, reducing 0.31 mM H_2_O_2_ at the same time (Figure 1B). Both modified GREs retained 100% activity in 4 consecutive cycles (total time of ca. 2 h). CAT-GRE was chosen for further experiments due to its higher activity. CPO immobilization on GRE was performed via electrochemically driven physical adsorption, followed by cross-linking with glutaraldehyde. The activity of immobilized CPO on GRE was investigated using the enantioselective transformation of thioanisol to (R)-methylphenylsulfoxide as a model reaction and with the addition of t-BuOH because of its hydroxyl radical scavenging properties, which improves the stability of CPO.^7^ On immersion of CPO-GRE in a solution containing 0.05 mM thioanisole and 0.1 mM H_2_O_2_, the production of methylphenylsulfoxide was observed (20 μM, 30 min) (Figure 1D) with retention of activity over a 2 h period (4 cycles). The immobilization of HRP on GRE was performed by electrochemically driven physical adsorption, followed by cross-linking. Upon immersion of the immobilized enzyme in solution (0.1 mM ABTS, 0.2 mM H_2_O_2_), the catalytic activity showed low stability (Figure 1C) and poor reproducibility over a period of 2 h (4 cycles). This likely arises from the leaching of HRP from GRE into the reaction solution, as evidenced by the continued production of ABTS^+^ after removing HRP-GRE from the solution (data not shown).

The stability of the enzyme-modified electrodes can be ascribed to the surface charge distribution of the enzymes. The isoelectric points of CAT, GOx, and CPO are ca. 5,^67^ 4.2,^27^ and 4,^68^ respectively. The surface charge distribution (Figure S2) of CAT, GOx, and CPO shows that the enzymes are negatively charged at pH 7. Application of a potential of 0.85 V to the electrode surface (graphite rod *E*_pzc_ of −0.237 V vs Ag/AgCl)^66^ results in an overall positive charge on the electrode surface, facilitating electrostatic interaction with the negatively charged enzymes. On the other hand, the poor stability of immobilized HRP is likely a result of its high PI 8.9^69^ with a positive surface charge distribution (Figure S2). While some HRP was adsorbed on the GRE, the overall negative surface charge distribution on HRP limits significant levels of adsorption onto the negatively charged electrode surface.

### 3.2 Flow Reactor

**Figure 2:**
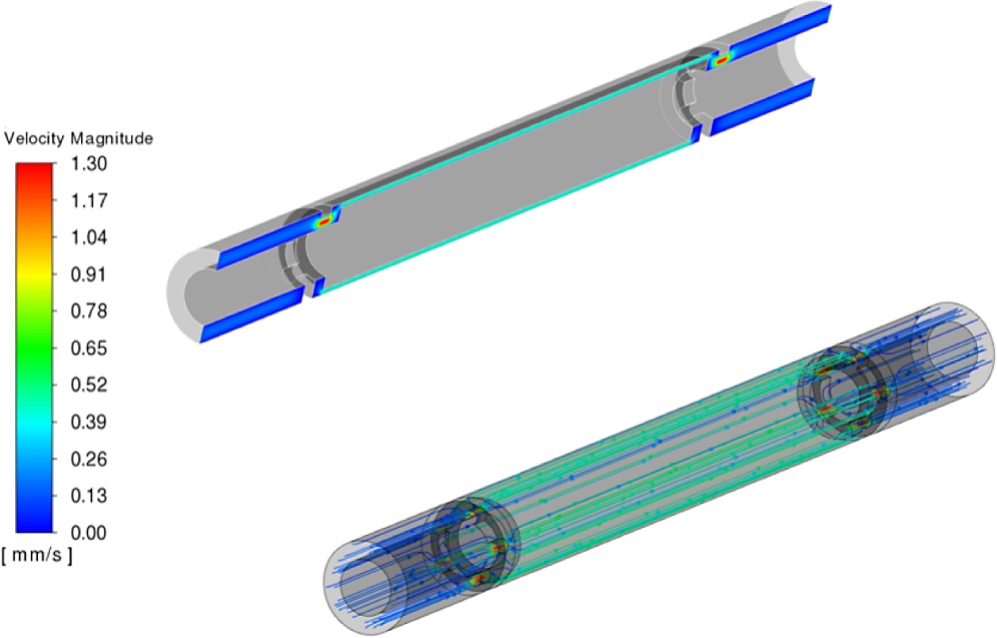
Simulated flow field of the reactor.

**Figure 3:**
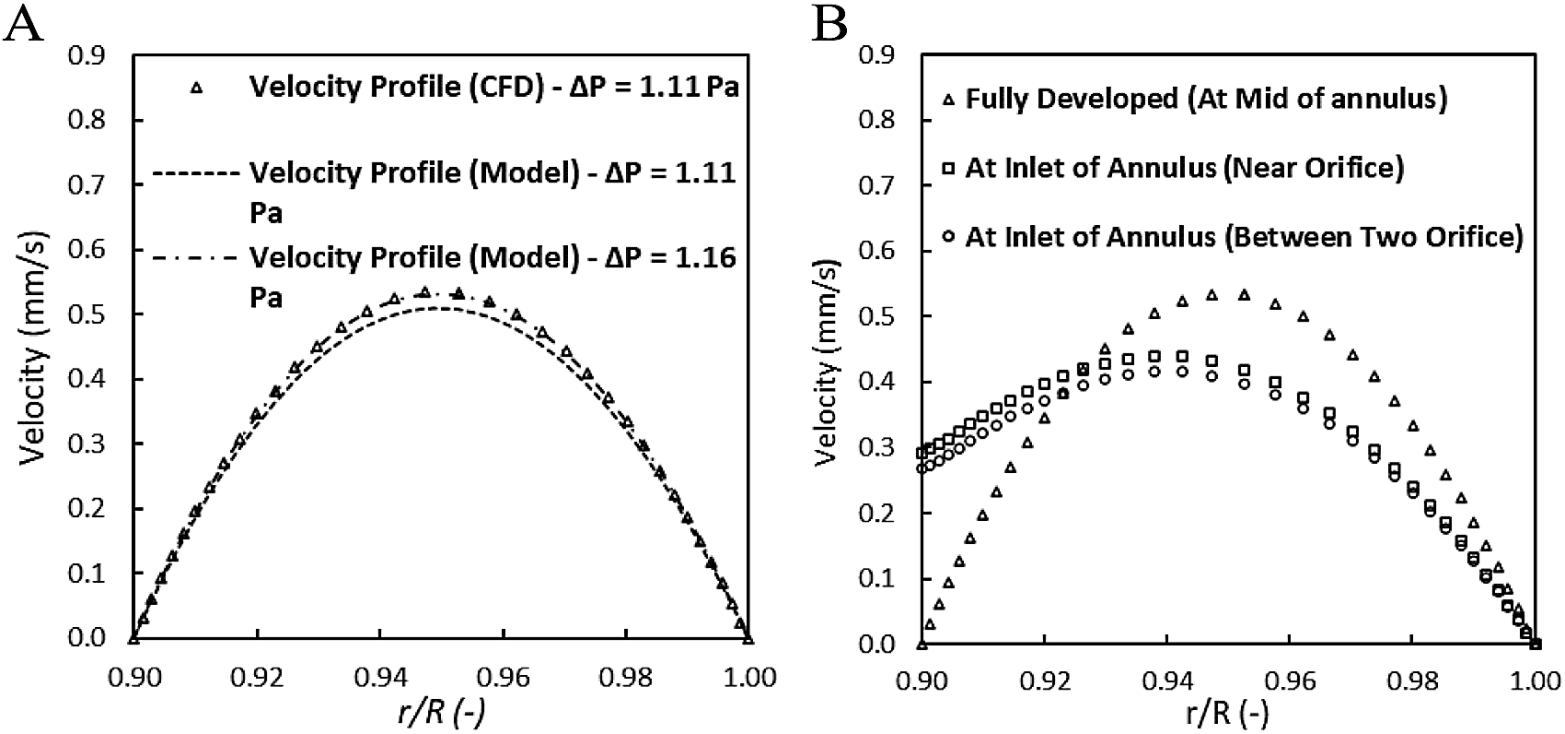
Comparison of radial velocity profiles in the annular reactor: (A) CFD and analytical solution for fully developed flow and (B) influence of end effects.

As immobilized enzymes were to be used in a flow system, a numerical analysis of the reactor was performed. Laminar flow and species transport in the reactor were characterized using a finite volume method^70−72^ (Supporting Information), to quantify the mixing and residence time distribution (RTD). The simulated flow field in terms of velocity contours and path lines is shown in Figure 2. The flow enters from the inlet surface and is subsequently distributed into five entrance nozzles (a diameter of 0.8 mm), with maximum fluid velocity occurring at the nozzles (Figure 2). The fluid then flows into the annular space, where it is fully developed in the annular space before exiting via five outlet nozzles. The flow pathlines (Figure 2) indicate that the flow pattern is well streamlined, typical of a laminar flow. Considering the narrow annular reactor, the velocity profile at the midplane may be approximated by a fully developed profile (eq 1):^73^
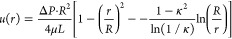
1where *u*(*r*) is the axial velocity at radial location *r* from the axis of the reactor, *ΔP/L* (Pa/m) is the pressure gradient at fully developed flow conditions, *R* is the radius of the reactor, and *κ* is the ratio of the radii of the inner and outer tubes. Figure 3A shows the velocity profile as a function of the nondimensional radius (*r*/*R*(-)). A value of 0.9 corresponds to a position on the inner wall of the annular space, that is the surface of the inner cylinder, while a value of 1 corresponds to the outer wall of the annular space (Figure S4). On comparison of the simulated velocity profile with the analytical solution (eq 1), the flow is fully developed at the midplane of the annular reactor (Figure 3A), which can be inferred from the characteristic parabolic velocity profile. However, the end effects caused by the five entrance and exit orifices influence the pressure drop and velocity profiles, as evidenced by the radial velocity profiles generated 0.1 mm before the inlet of the annulus (Figure 3B).

**Figure 4:**
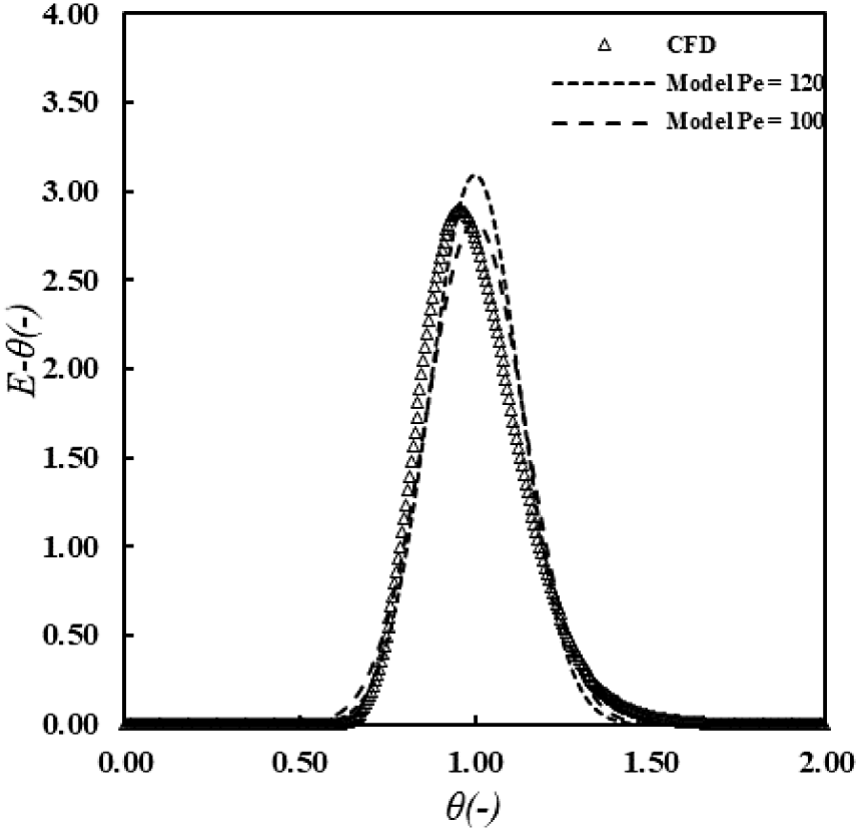
Residence time distribution of the annular reactor.

It is important to quantify the residence time distribution (RTD) of the annular reactor in order to develop a model successfully. RTD was simulated via the step input of a tracer, and the concentration of the tracer was monitored at the outlet of the reactor to determine the RTD by established standard procedures^74−76^ (the detailed numerical process is provided in Supporting Information). The RTD of *t*(*E* – θ(-) vs θ(-)) was obtained (Figure 4), where *E* – θ(-) is the nondimensional exit age distribution function and θ(-) nondimensional time calculated as  (*t* is flow time and  is mean residence time—Supporting Information). The Péclet number (—ratio of convective transport rate to diffusive transport rate) obtained by CFD simulation was ∼ 120, where *D*/*uL* is the vessel dispersion number (-). The RTD obtained from CFD simulation was compared with the analytical solution given by Levenspiel^77,78^ for the axial dispersion model, for a low dispersion number (<0.01) (eq 2). The Péclet number from CFD was ∼120 while the analytical solution with Péclet number = 100 had a close match with the CFD. This can be attributed to a slight increase in the convective component of the flow due to inlet orifices. Overall, very little difference was seen between the RTD obtained by CFD and the observed analytical solution (Figure 4).
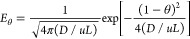
2

**Figure 5:**
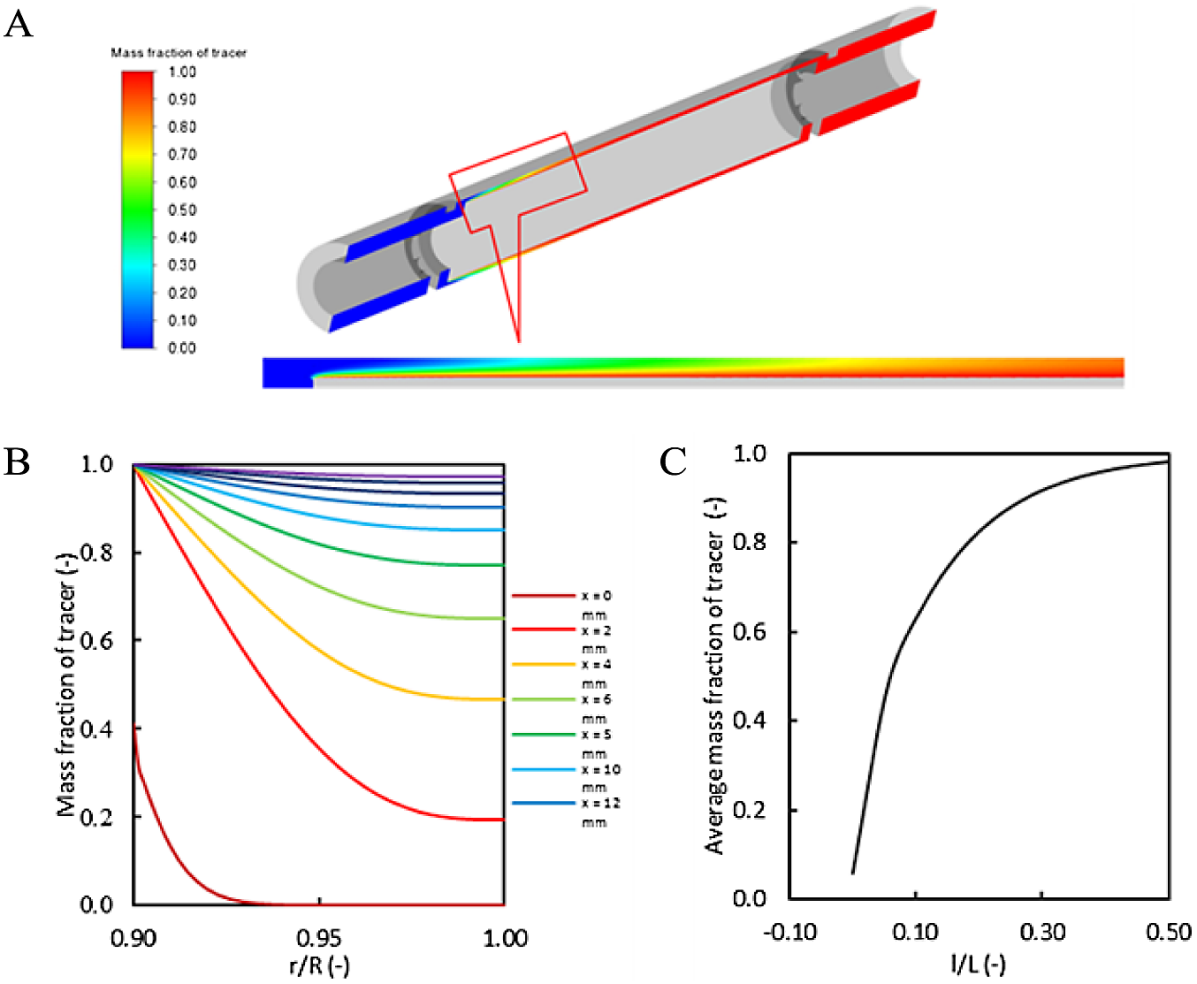
Spread of species generated at the surface of the inner cylinder: (A) contour of the mass fraction of tracer over the midplane in the annular region; (B) profile of mass fraction over a radial line in the annular region (from the inlet of the annular chamber to the middle of the chamber); (C) average mass fraction of the tracer as a function of dimensionless axial distance.

The model was then used to understand the mixing of species generated at the outer surface of the inner cylinder due to the enzymatic reaction. The diffusion of species generated at the surface was simulated by specifying an arbitrary mass fraction of the tracer at the surface of the inner cylinder (Figure 5A), generating a profile of the mass fraction on the radial monitoring line (Figure 5B). Mass fraction profile lines were generated from the inlet of the annular space where the inner cylinder begins up to the middle length of the cylinder (Figure 5B). The average mass fraction becomes uniform (Figure 5C), attaining a value of ∼0.99 in the middle of the reactor, indicative of complete mixing. The effective Péclet number was in the order of 10^2^, and therefore, an approximation of small deviation from the ideal plug flow reactor is reasonable. The CFD model demonstrates that the length of the reactor and gap between the outer and inner cylinder are appropriate for the reactions under consideration.

The scope of this work is restricted to reporting the overall performance of this well-characterized annular reactor for the three-enzymatic cascade reactor for peroxidasease-catalyzed reactions. The development of detailed reactor models integrating flow, mixing, and intrinsic kinetics will be presented separately.

### 3.3 GOx-GRE, CAT-GRE, and CPO-GRE in the Flow Reactor

**Figure 6:**
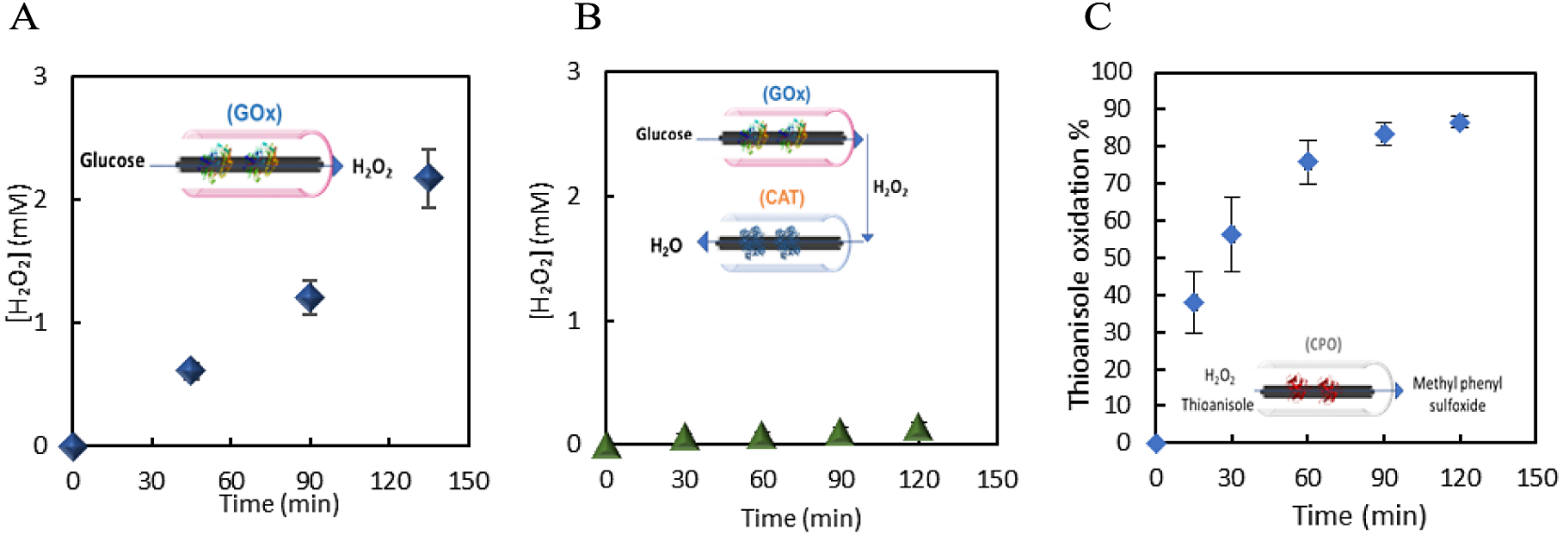
Plots of (A) concentration of H_2_O_2_ produced at GOx-GRE (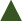); (B) concentration of H_2_O_2_ produced in bienzymatic cascade GOx/CAT-GRE (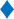) in the flow reactor; 50 mM glucose in NaPi (0.1 M, pH 5), flow rate of 0.08 mL min^–1^ and total volume of 3 mL; (C) conversion (%) of thioanisole at CPO-GRE (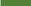) in the flow reactor; 50 mM glucose, ca. 0.1 mM, thioanisole, in 20% t-ButOH: NaPi (0.1 M, pH 5), flow rate of 0.08 mL min^–1^ and total volume of 3 mL. Error bars indicate the standard deviation of triplicate experiments.

The performance of the modified GREs was investigated in a flow reactor at a flow rate of 0.08 mL min^–1^ and a total volume of 3 mL in a closed loop. GOx-GRE was incorporated into the reactor (Figure 6A) in a solution containing 50 mM glucose and 1 and 2 mM of H_2_O_2_ was produced after 1 and 2 h, respectively (24-fold higher than when polypyrrole was used as a support).^26^ When GOx-GRE was subsequently combined with CAT-GRE (Figure 6B), the concentration of H_2_O_2_ after 1 h was 0.074 mM, demonstrating that immobilized catalase effectively catalyzes the decomposition of H_2_O_2_ (Figure 6B). After two hours of operation, the concentration of H_2_O_2_ in the GOx/CAT-GRE system was 0.15 mM, demonstrating that CAT-GRE efficiently removed >93% of the H_2_O_2_ produced by GOx-GRE, and that the two-enzyme system operated successfully in the flow reactor. The final step in the formation of the cascade was the addition of immobilized CPO, which oxidizes indole using *in situ* generated H_2_O_2_. However, the poor solubility of indole in aqueous media requires the addition of an organic solvent.^15^ t-BuOH was selected as solvents; in addition to solubilizing indole, it acts as a scavenger of hydroxyl radicals, improving the stability of CPO.^3^ The activity of CAT-GRE and GOx/CAT-GRE was investigated in the flow system in the presence of 20% t-BuOH (Figure S1). GOx-GRE showed higher activity (16%) in the presence of t-BuOH, with a 1.25 mM H_2_O_2_ produced after 1 h (Figure S1). This increase can be attributed to the radical scavenging abilities of t-BuOH, which effectively preserve the activity of GOx. When GOx-GRE was combined with CAT-GRE, the concentration of H_2_O_2_ was 1 mM after 2 h (Figure S1), while the GOx-GRE system generated 2.5 mM H_2_O_2_ after 2 h. The results demonstrate that CAT-GRE removed only 60% of the H_2_O_2_ produced by GOx-GRE in the presence of t-BuOH, in contrast to >93% removal in the absence of t-BuOH. The activity of catalase has been shown to be consistently lower in water–alcohol mixtures in comparison to aqueous solution.^79^ The impact of alcohols on the rate of the catalase catalyzed decomposition of hydrogen peroxide arises from the interplay between solute entities (enzyme, substrate, enzyme–substrate complex, and transition states) and the solvent, with solvent properties such as solvent hydrophobicity and dielectric constant affecting the catalytic activity of the enzyme.^79^

In order to improve the efficiency of H_2_O_2_ removal, the concentration of CAT in the immobilization solution was increased from 2 to 4 mg mL^–1^. As expected, GOx/CAT (4 mg mL^–1^ CAT)-GRE produced 0.65 mM H_2_O_2_ after 2 h (Figure S1), which is lower than the concentration of H_2_O_2_ (1 mM) produced in GOx/CAT-GRE flow system where 2 mg mL^–1^ CAT was employed for electrodeposition (Figure S1). The results demonstrate that CAT (4 mg mL^–1^ CAT)-GRE removed 74% of the H_2_O_2_ produced by GOx-GRE in the presence of t-BuOH, a satisfactory result. It should be noted that it is not necessary to remove all the H_2_O_2_ as much of it will be used by the CPO or HRP. Before the target cascade was created, the activity of CPO-GRE was investigated in a solution of 0.1 mM thioanisole and 0.2 mM H_2_O_2_ in the presence of t-BuOH (Figure 6C). CPO-GRE oxidized 87% of the thioanisole to the corresponding sulfoxide after 2 h of reaction.

**Scheme 3:**
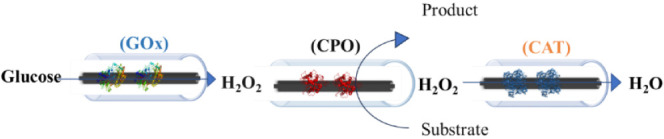
Schematic Diagrams of a Three-Enzyme Cascade in a Flow System Using Immobilized CPO on GRE Each enzyme was immobilized onto an individual graphite rod, subsequently interconnected to form a series of three reactors.

Based on these results, a three-enzyme system, GOx-GRE, CPO-GRE, and CAT-GRE, was examined in the oxidation of indole to 2-oxindole (Scheme 3).

Utilizing a three-enzyme cascade system GOx/CPO/CAT-GRE, in the presence of a solution containing 1 mM indole and 50 mM glucose, a conversion of 36% of indole to 2-oxindole was achieved following 4 h of continuous reactor operation (Figure S3). The efficacy of *in situ* generation of H_2_O_2_ by the entire cascade (GOx/CPO/CAT-GRE) was compared with the activity of CPO-GRE obtained by external addition of H_2_O_2_. A low rate of conversion (7.2%) of indole to 2-oxindole (Figure S3 purple) was obtained with the latter, in contrast to 36% for the former. The results obtained clearly show that a single addition of a relatively high concentration of H_2_O_2_ (1 mM) has a negative effect on the enzyme activity. It was therefore decided that H_2_O_2_ would be added in four smaller doses (250 μM) at 0, 30, 60, and 90 min. Again, the efficiency of CPO-GRE was low; only 8.4% of indole was converted to 2-oxindole. In addition, the decrease in the concentration of indole was greater than the increase in the concentration of 2-oxindole (data not shown), indicating that additional side reactions had occurred. External addition of H_2_O_2_, even in small portions, negatively affects the efficiency of 2-oxindole formation in annular flow reactors. Due to the small volume of the reactors, the addition of H_2_O_2_ could lead to a local increase in the concentration of this reactant, which would have a negative effect on the activity of CPO, highlighting the importance of combining GOx and CAT with CPO.

Immobilized enzymes (GOx, CAT, and CPO) showed good stability in the batch system (Figure 1); however, in the flow system, the enzymes are exposed to shear forces that can cause deactivation or leaching, which might be the reason for lower oxidation indole oxidation conversion. To better understand this phenomenon, the stability of the individual immobilized enzymes in the flow system was examined.

**Figure 7:**
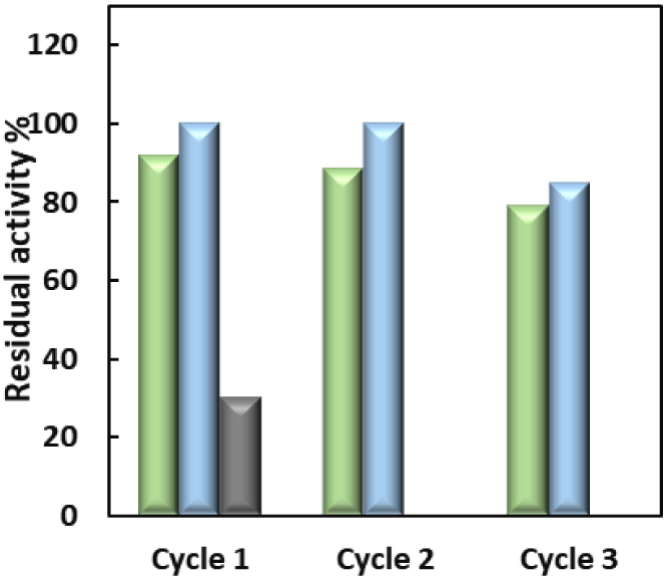
Operational stability of CAT-GRE (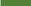), GOx-GRE (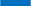), and CPO-GRE (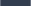) in the flow reactor. The activity of each immobilized enzyme was determined prior to insertion into the reactor and used as the baseline activity.

The operational stabilities of CAT-GRE and GOx-GRE were investigated over three reaction cycles, each of 2 h duration (Figure 7). CAT-GRE and GOx-GRE retained ca. 79% and 84% of their initial activity, respectively. The observed decrease in the activity of CAT-GRE and GOx-GRE can be attributed to either enzyme leaching from the electrode surface to the solution or enzyme inactivation by H_2_O_2_. As the concentration of H_2_O_2_ was less than 0.15 mM, the decrease likely arises from leaching of the enzyme.

When the operational stability of CPO-GRE was investigated with a solution of 0.1 mM thioanisole and 0.2 mM H_2_O_2_ in the presence of t-ButOH (Figure 7), CPO-GRE exhibited a significant decrease in activity, retaining only 30% of its initial level after the first cycle, with no activity observed in subsequent cycles. When a solution containing only buffer was used in the flow reactor (2 h), the activity decreased by 70%, indicating that the enzyme was leaching from the surface (results not shown). The findings indicate that the immobilization method was successful for CAT and GOx, but not for CPO. Following the investigation of the stability of immobilized enzymes within the system, it is evident that the low conversion of indole to 2-oxindole in the three-enzymatic cascade (GOx/CPO/CAT-GRE) within the flow reactor can be ascribed to the leaching of CPO from the graphite surface.

Due to the low operational stability of immobilized CPO in the flow system, free CPO was used with GOx/CAT-GRE to perform the oxidation of indole to 2-oxindole and the halogenation of the phenolic monoterpenes, thymol and carvacrol to their corresponding halogenated products.

**Figure 8:**
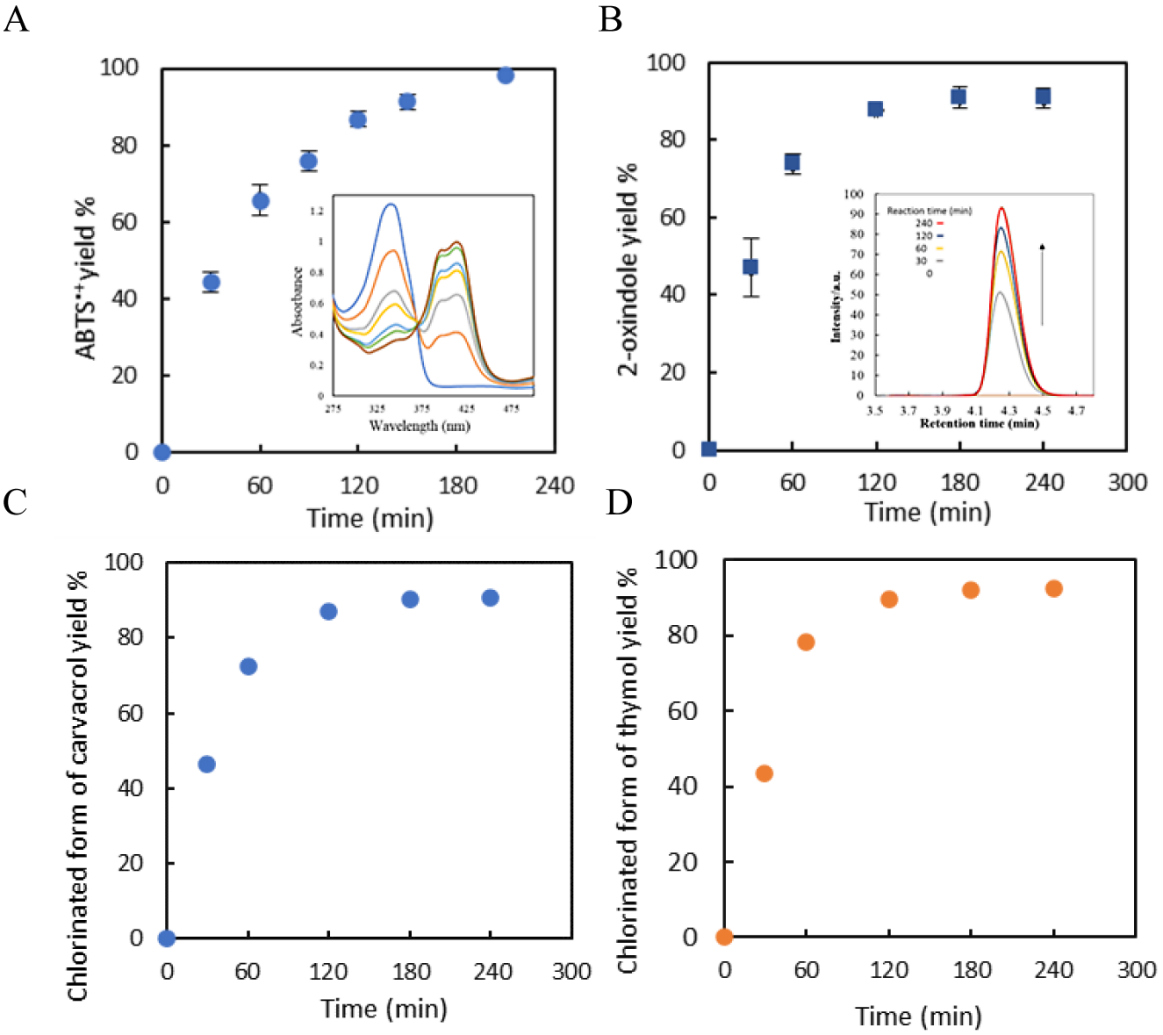
Plots of (A): ABTS oxidation in the three enzyme cascade GOx/free HRP/CAT-GRE in the flow reactor; inset: the absorbance spectra of ABTS and ABTS^+^ in the reactor; 50 mM glucose, 1 mM ABTS, 0.2 mg ml^–1^ HRP in NaPi (0.1 M, pH 5), a flow rate of 0.08 mL min^–1^ and a total volume of 3 mL; (B): %yield of 2-oxindole in three-enzyme enzymatic cascade (GOx/free CPO/CAT-GRE) in the flow reactor; inset: HPLC chromatograms recorded for 2-oxindole; 50 mM glucose, ca. 1 mM, indole, 8 μg ml^–1^ CPO in 20% t-ButOH: NaPi (0.1 M, pH 5), a flow rate of 0.08 mL min^–1^ and a total volume of 3 mL; (C): the chlorination of (C) carvacrol and (D) thymol in the three-enzyme enzymatic cascade (GOx/free CPO/CAT-GRE) in the flow reactor. 50 mM glucose, ca. 1 mM, carvacrol or thymol, 8 μg ml^–1^ CPO in 20% t-ButOH: NaPi (0.1 M, pH 5), a flow rate of 0.08 mL min^–1^, and a total volume of 3 mL. Error bars indicate the standard deviation of duplicate experiments (*n* = 2).

The selective oxidation of 1 mM indole to 2-oxinolde was performed using (GOx-GRE/free CPO (0.023 mg = 17 U)/CAT-GRE) in 20% t-ButOH. Under these conditions, 74% of indole was oxidized into 2-oxindole in 60 min, increasing to 91% in 180 min (Figure 8B). The TON, TOF, and STY values were 5456 (mol product/mol enzyme^–1^), 30.3 (min^–1^), and 0.97 (g L^–1^ d^–1^), respectively. The halogenation of thymol and carvacrol was investigated using (GOx-GRE/free CPO/CAT-GRE) in the flow system in a solution of 1 mM thymol or carvacrol and 20 mM sodium chloride in 20% t-BuOH. (Figure 8C,D). The reaction was carried out for 4 h, resulting in 91% and 93% conversion of carvacrol and thymol, respectively, to their corresponding halogenated product. LC-QTOF analysis of the reaction products confirmed the formation of dichlorocarvacrol and a mixture of chorothymol and dichlorothymol (Figures S6-S9).

To explore the broad applicability of *in situ* H_2_O_2_ generation, GOx/CAT-GRE, along with widely used peroxidase enzymes, were employed in a flow system. In this setup, HRP (in solution) was combined with GOx/CAT-GRE to catalyze the oxidation of ABTS, serving as a model reaction (see Figure 8A). The oxidation of ABTS to ABTS^+■^ was complete in 210 min, demonstrating that GOx/CAT-GRE can be successfully combined with peroxidases for oxidation reactions.

## 4 Conclusion

The enzyme cascade system described enables the controlled delivery of H_2_O_2_, with the potential to overcome the problems associated with the low stability of peroxygenases in the presence of H_2_O_2_. The flow reactor exhibited laminar flow properties, and detailed characterization indicated that uniform flow had developed in the reactor. When GOx and CAT were immobilized on a graphite rod via electrochemically driven adsorption together with cross-linking with glutaraldehyde, the immobilized enzymes showed good stability, retaining 84% and 79% of their initial activity, respectively, after 3 cycles of operation in the flow reactor. However, immobilized CPO exhibited a considerable reduction in activity after a single use, suggesting the need for further optimization in its immobilization process. A GOx/CAT-GRE reactor provided an effective way of delivery of H_2_O_2_ to chloroperoxidase, resulting in a 4-fold enhancement in the yield of oxyindole compared to the direct addition of H_2_O_2_. The capability of the GOx/CAT-GRE system, coupled with free CPO, was demonstrated by achieving high yields (>90%) for the oxidation of indole to 2-oxindole and the chlorination of thymol and carvacrol. In conclusion, this study highlights the potential of electrochemical methods of immobilizing enzymes in flow reactors. The experimental kinetic data can be combined with CFD modeling to gain better insights into understanding the effects of flow rate, mixing and residence time distributionon on the reaction conversion and yield and will be used to provide a better platform to develop a comprehensive reactor model.

## Supporting Information Available

Surface charge distribution of enzymes, flow modeling of the annular reactor, the activity evaluation of GOx-GRE, CAT/GOx in the flow system in t-ButOH, selective oxidation of indole to 2-oxindole, and at three enzymatic cascade GOx/CPO/CAT-GRE (PDF)
