# Supplementary material for: Controlled Delivery of H2O2: A Three-Enzyme Cascade Flow Reactor for Peroxidase-Catalyzed Reactions
Source: ACS Sustain Chem Eng. 2024 Jun 27;12(28):10555–66. doi: 10.1021/acssuschemeng.4c03220 (PMC11253098; doi:10.1021/acssuschemeng.4c03220)
Supplement: Supplementary file 1 — sc4c03220_si_001.pdf [file sc4c03220_si_001.pdf]

Controlled Delivery of H_2O_2: A Three Enzyme Cascade Flow Reactor for Peroxidase-Catalyzed Reactions

Simin Arshi[a], Ketan Madane[a], Kim Shortall[a], Goran Hailo[a], Julia Alvarez-Malmagro[a], Xinxin Xiao[b], Katarzyna.Syzmanńska[c], Serguei Belochapkine[a], Vivek V. Ranade[a], Edmond Magner[a]*

Supporting Information

Table of Contents

Figure S1. Plot of concentration of H_2O_2 produced at GOx-GRE. S2

Figure S2. Surface charge distribution of GOx, HRP, CPO, and CAT. S2

Figure S3. Plots of selective oxidation of indole to 2-oxindole. S3

Figure S4: Dimensions of the electrochemical reactor. S4

Figure S5: Mesh at different locations. S8

Figure S6. HPLC chromatogram and MS spectrum of thymol. S8

Figure S7. HPLC chromatogram and MS spectrum of chlorothymol. S8

Figure S8. HPLC chromatogram and MS spectrum of carvacrol. S8

Figure S9. HPLC chromatogram and MS spectrum of dichlorocarvacrol S9

Flow modeling of annular reactor. S3

1. Geometry S3
2. Computational Modelling S4

References S9

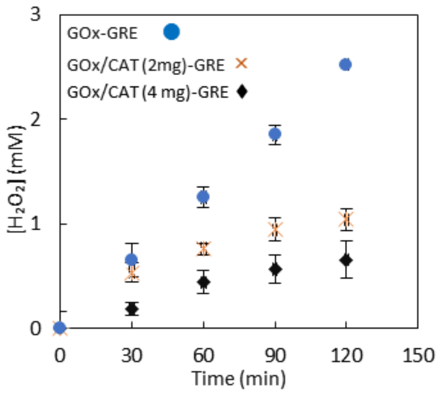

Figure S1. Plot of concentration of H_2O_2 produced at GOx-GRE in the presence of t-ButOH (●), bi-enzymatic cascade GOx/CAT-GRE (CAT, 2 mg ml^{-1}) (×) and GOx/CAT-GRE (CAT, 4 mg ml^{-1}) (♦) in the reactor, 50 mM glucose in NaPi (0.1 M, pH 5), flow rate of 0.08 ml min^{-1} and total volume of 3 ml. Error bars indicate the standard deviation of duplicate experiments (n = 3).

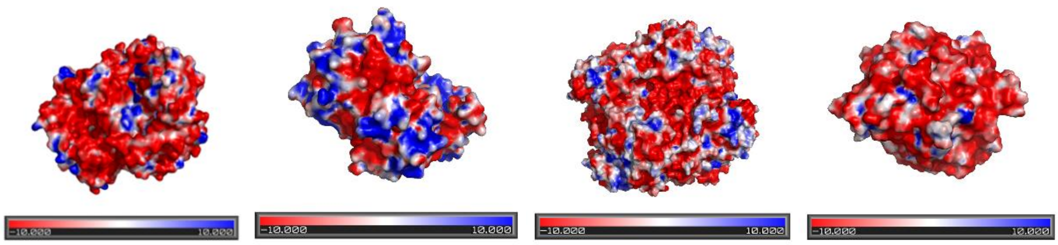

Figure S2. Surface charge distribution of (left to right) GOx (PDB: 1CF3), HRP (PDB: 2YLJ), CPO (1CPO), and CAT (PDB: 1TGU) obtained using PyMOL [1] at pH 7.0 with positive and negative charges indicated by blue and red colors, respectively.

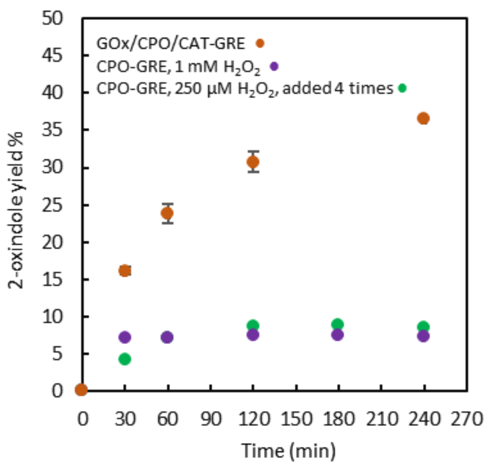

Figure S3. Plots of selective oxidation of indole to 2-oxindole; at three enzymatic cascade GOx/CPO/CAT-GRE (●); at CPO-GRE in the presence of 1 mM H_2O_2 (●), at CPO-GRE with the addition of H_2O_2 0.25 mM, 4 times at 0 min, 30 min, 60 min, and 90 min (●), in the flow reactor. 50 mM glucose, ca. 1 mM, indole in 20% t-ButOH:NaPi (0.1 M, pH 5). Error bars indicate the standard deviation of duplicate experiments (n = 2)

Flow modeling of annular reactor

1. Geometry

The geometry of the reactor is a tubular annular reactor which has an annular inlet of 2mm that converges into 5 orifices of 0.8 mm diameter. The outlet of the 5 orifice opens in an annular spacing of 0.25 mm that runs for 36 mm axially. The reactor geometry is symmetric about the normal axis of the geometry. The geometry considered in this study is shown in the figure 3-1.

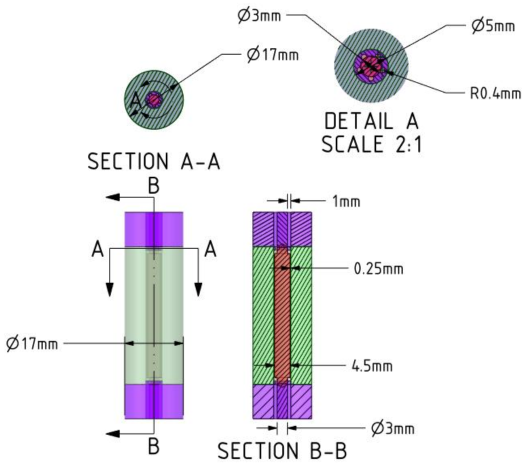

Actual design of the reactor

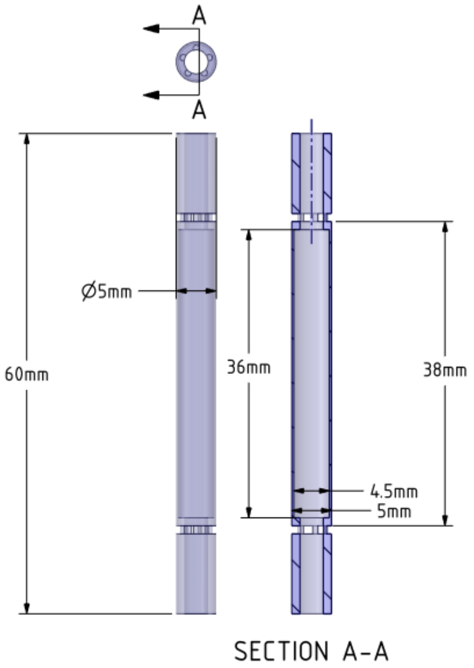

Fluid volume considered for simulation

Figure S4: Dimensions of the electrochemical reactor

2. Computational Modelling

a. Model Equations

For the considered design and the operating flow rate of 0.08 mL/min, the inlet Reynolds number ($Re = \frac{\rho v d_h}{\mu}$ where ρ is the density of the fluid in kg/m^3, v is the mean velocity in m/s, d_h is the hydraulic inlet diameter in m [$d_h = d_o - d_i$], and μ is the dynamic viscosity Pa.s) was found to be of 0.1. The maximum Reynold number 0.65 was found to be in the orifice. Based on the calculated Reynolds number the flow in the reactor was in the creeping regime ($Re < 1$). The Knudsen number (Kn) was also calculated to check the validity of fluid continuum ($Kn < 0.01$). The value Kn of was found to be in the order of 10^{-7}. As the calculated Reynolds number was not in the turbulent regime ($Re > 2000$), the flow in the reactor was modelled with the help of Laminar flow model. The following governing equation viz. Continuity and momentum were solved to simulated the flow in the reactor.

$$\nabla \cdot (\vec{v}) = 0 \quad (1)$$

$$\frac{\partial}{\partial t}(\rho \vec{v}) + \nabla \cdot (\rho \vec{v} \vec{v}) = -\nabla p + \nabla \cdot (\bar{\bar{\tau}}) \quad (2)$$

Where $\vec{v}$ is the Reynolds-averaged velocity vector (m/s), ρ is the density (kg/m^3), p is the static pressure (N/m$^{2)}$, t is time (s), $\bar{\bar{\tau}}$ is the effective stress tensor (N/m^2).

For the residence time distribution (RTD) following species transport equation was used to simulate multispecies flow

$$\frac{\partial(\rho m_k)}{\partial t} + (\vec{v} \cdot \nabla) m_{km} = D_{km} \nabla^2 m_k \quad (3)$$

where, m_{km} is the mass fraction of the tracer of species k and D_{km} is the mass diffusion coefficient of the species k in the mixture. The viscosity and the mass diffusivity of the tracer in the mixture were considered constant. For the determining the RTD of the reactor, the tracer was numerically injected from the inlet surface. It was ensured that more than 99.8% per cent of the injected tracer has passed through the outlet. The RTD was also determined separately by giving specifying mass fraction a on the inner wall of the mid annular space.

b. Boundary conditions and post processing

The flow and residence time distribution (RTD), simulations were performed in the geometry shown in Figure 1 for a flow rate of 0.08mL/min. Velocity was calculated based on the inlet annular area and the volumetric flow rate. The calculated velocity magnitude was given as flat velocity inlet boundary condition to the inlet surface of the reactor. The direction of the velocity was normal to the inlet surface. The outlet surface with the same dimensions as the inlet surface was defined as a pressure outlet (constant pressure boundary condition).

Water was used as a working fluid with a constant density (ρ) of 1000 kg/m^3, and the dynamic

viscosity (μ) of 0.001 Pa.s. For the RTD simulation of the secondary species, the tracer was assumed to have the same properties as water. The mass diffusivity of the tracer was assumed constant with a magnitude of 2 x 10^{-9} m^2/s.

For the RTD study, simulated tracer response after a step input was analyzed using the classic tracer theory presented by Levenspiel[2, 3]. The mean average residence time $\bar{t}$ (s), the variance of the curve σ^2, E – θ curve and the Dispersion number (D/uL) (where D is the dispersion coefficient), were calculated as:

$$\bar{t} = \frac{\int_0^\infty t\mathrm{m_i}dt}{\int_0^\infty \mathrm{m_i}dt} \cong \frac{\sum_i t_i \overline{\mathrm{m_i}}_i \Delta t_i}{\sum_i \overline{\mathrm{m_i}}_i \Delta t_i} = \frac{\sum t_i \overline{\mathrm{m_i}}_i}{\sum \overline{\mathrm{m_i}}_i} [s] \tag{4}$$

$$\sigma^2 = \frac{\sum t_i^2 \mathrm{m_i}_i \Delta t_i}{\sum \mathrm{m_i}_i \Delta t_i} - \bar{t}^2 = \frac{\sum t_i^2 \mathrm{m_i}}{\sum \mathrm{m_i}} = \bar{t}^2 [s^2] \tag{5}$$

$$\sigma_\Theta^2 = \frac{\sigma^2}{\bar{t}^2} \cong 2\frac{\Gamma}{uL} \tag{6}$$

$$E = \frac{\mathrm{m_i}}{\int_0^\infty \mathrm{m_i}dt} = \frac{\mathrm{m_i}}{\mathrm{m_i}t}\left[\frac{1}{s}\right] = s^{-1} \tag{7}$$

$$E_\theta = \bar{t}E[-] \tag{8}$$

Where $\mathrm{m_i}$ is the mass fraction of the tracer (-), and. $\theta = t/\bar{t}$ (-) is the non-dimensional mean residence time, and E is the mean residence time distribution function (s^{-1}).

$$\boldsymbol{E_\theta} = \frac{1}{\sqrt{4\pi(\boldsymbol{D}/uL)}} \exp\left[-\frac{(1-\theta)^2}{4(\boldsymbol{D}/uL)}\right]$$

c. Solution to model equation

The model equations were numerically solved by the finite volume method[4-7] using the

commercial CFD code Ansys Fluent (Ansys Inc, Version 2021 R2). The governing equations were spatially discretized using the Third-order MUSCL scheme. The pressure and velocity coupling were considered using the SIMPLE algorithm. The pressure was discretized using a PRESTO! scheme. The under relaxation factors were 0.3 and 0.7 for pressure and momentum, respectively. For flow simulation steady state simulation was performed. All the parameter were converged to 10^{-6}.

For residence time distribution simulation, converged flow field from the flow simulation was used and only species transport equations were solved. The convergence criteria of 10^{-7} were met for the species. Simulation for flow time of 5τ was sufficient to reach a flat mass-weighted averaged concentration of tracer at the outlet of the reactor. The time step was calculated by generally accepted thumb rule of $\frac{\tau}{1000}$ The simulated outlet concentration data were collected and processed for the RTD curve (E) and the Dispersion number (D/uL) according to Equations 4-8.

d. Mesh

The 3-dimensioanl mesh was used for the numerical simulation was generated in Ansys meshing platform. This was strategically split into sweepable blocks in Ansys SpaceClaim to accommodate only hexahedral and pave elements in the entire fluid volume. The curvature and proximities were captured and were also scoped critically, to ensure high quality elements in the critical regions. Biasing was given to critical edges and faces to ensure appropriate growth of cells and transition of cell size. Figure 3-2 shows the mesh images at critical locations.

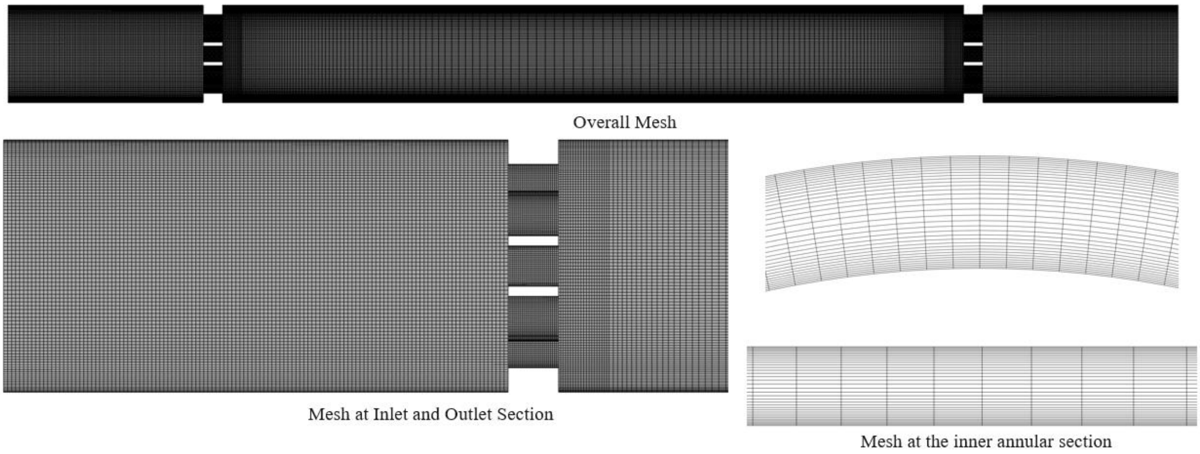

Figure S5: Mesh at different locations

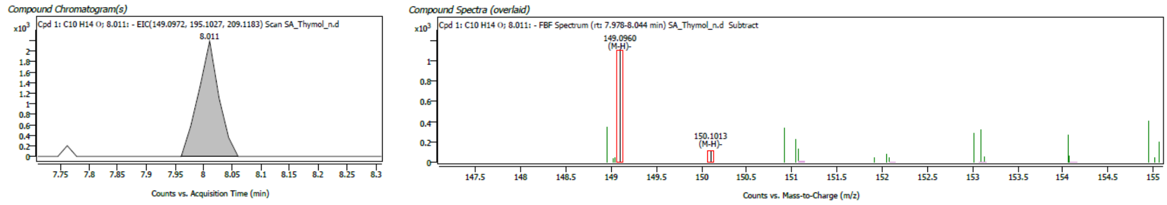

Figure S6. HPLC chromatogram and MS spectrum of thymol

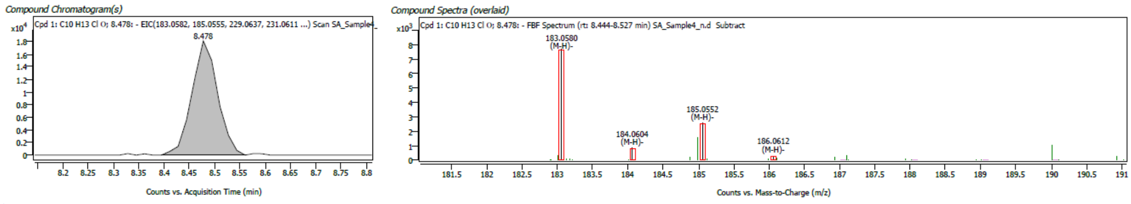

Figure S7. HPLC chromatogram and MS spectrum of chlorothymol

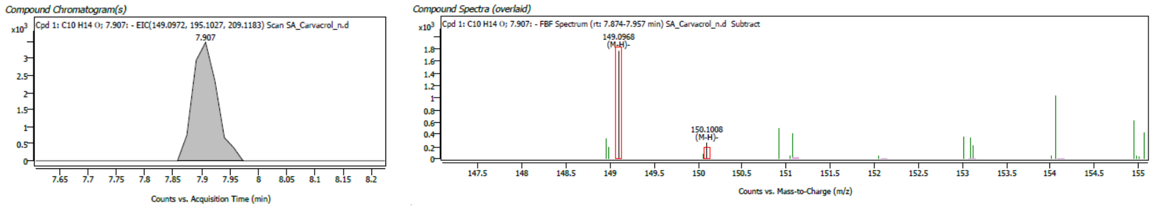

Figure S8. HPLC chromatogram and MS spectrum of carvacrol

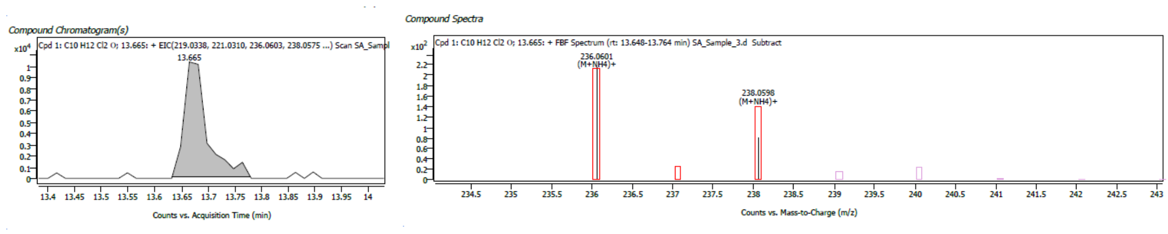

Figure S9. HPLC chromatogram and MS spectrum of dichlorocarvacrol

References

(1) DeLano, W. PyMOL 0.99. rc6 Palo Alto. *CA, USA* **2002**.
(2) Levenspiel, O. *Chemical reaction engineering*; John wiley & sons, 1998.
(3) Levenspiel, O. *Tracer technology: modeling the flow of fluids*; Springer Science & Business Media, 2011.
(4) Patankar, S. *Numerical heat transfer and fluid flow*; Taylor & Francis, 2018.
(5) Ranade, V. V. *Computational flow modeling for chemical reactor engineering*; Academic press, 2002.
(6) Anderson, J. D.; Wendt, J. *Computational fluid dynamics*; Springer, 1995.
(7) Date, A. W. *Introduction to computational fluid dynamics*; Cambridge University Press, 2005.
